# GnRH peripherally modulates nociceptor functions, exacerbating mechanical pain

**DOI:** 10.3389/fnmol.2024.1160435

**Published:** 2024-05-09

**Authors:** Haiyan Zheng, Minseok Kim, Chaeun Kim, Yerin Kim, Pyung Sun Cho, Ji Yeon Lim, Hojin Lee, Hye-In Yun, Jungmin Choi, Sun Wook Hwang

**Affiliations:** Department of Biomedical Sciences, Korea University College of Medicine, Seoul, Republic of Korea

**Keywords:** GnRH, GNRHR, pain, neuropathic pain, DRG neuron

## Abstract

The function of peripheral nociceptors, the neurons that relay pain signals to the brain, are frequently tuned by local and systemic modulator substances. In this context, neurohormonal effects are emerging as an important modulatory mechanism, but many aspects remain to be elucidated. Here we report that gonadotropin-releasing hormone (GnRH), a brain-specific neurohormone, can aggravate pain by acting on nociceptors in mice. GnRH and GnRHR, the receptor for GnRH, are expressed in a nociceptor subpopulation. Administration of GnRH and its analogue, localized for selectively affecting the peripheral neurons, deteriorated mechanical pain, which was reproducible in neuropathic conditions. Nociceptor function was promoted by GnRH treatment *in vitro*, which appears to involve specific sensory transient receptor potential ion channels. These data suggest that peripheral GnRH can positively modulate nociceptor activities in its receptor-specific manner, contributing to pain exacerbation. Our study indicates that GnRH plays an important role in neurohormonal pain modulation via a peripheral mechanism.

## 1 Introduction

Peripheral nociceptors of dorsal root ganglia (DRG) monitor potential tissue damage and transduce and convey such information as pain signals (Hwang and Oh, 2007; Dubin and Patapoutian, 2010). G-protein coupled receptors (GPCRs) for neurotransmitters, neurohormones, and pro- and anti-inflammatory mediators are expressed in these neurons and tune their excitability when stimulated by ligands (Hanack et al., 2015; Choi et al., 2016; Choi and Hwang, 2016; Choi and Hwang, 2018; Cho et al., 2021; Zheng et al., 2021). Peptidergic neurohormones are considered among the important ligands playing a role in this fashion. Endogenous opioids and oxytocin are the best examples that work through descending modulatory axons mainly from periaqueductal gray, rostral ventromedial medulla, and paraventricular nuclei (Corder et al., 2018; Zheng et al., 2021). Besides such circuit-based mechanisms, the evidence for independent productions in the periphery including immune cells or nociceptors themselves and for modulatory mechanisms in paracrine and autocrine manners are currently increasing (Pinho-Ribeiro et al., 2017; Zheng et al., 2020). Pathologic leaks owing to the breakdown of blood–brain barriers (BBBs) that normally secure neurohormones inside the brain or exaggerated increases in hormonal levels may be additional causes that allow those substances to reach nociceptors at an effective concentration (Edvinsson et al., 2019). Nonetheless, compared to the large pool of these brain-specific and peptidergic hormones, only limited information is available about whether and how variable each of the neurohormones affects nociceptor functions and resultant outcomes are.

Gonadotropin-releasing hormone (GnRH) is a hypothalamic peptidergic hormone that stimulates the anterior pituitary and initiates the hypothalamic–pituitary–gonadal axis. Mild expressions of GnRH and its receptor in the spinal cord have been observed but they were not readily detected in a descending terminal potentially from higher brain regions (Dolan et al., 2003; Quintanar et al., 2009; Dïaz-Galindo et al., 2021). On the other hand, the receptors seem to be expressed in DRG neurons according to a recent series of massive transcriptomic analyses (Usoskin et al., 2015; Kupari et al., 2021). Interestingly, in the clinical settings for treating hormone-dependent cancers or endometriosis, GnRH analogues often lead to peripheral adverse effects in patients including pain, redness, and swelling at their injection sites (Kuhn et al., 2016). Accordingly, we considered whether interactions of GnRH and its receptor GnRHR may occur in nociceptors and alter neuronal function and by this peripheral mechanism, pain may be induced and/or aggravated.

## 2 Results

### 2.1 GnRH and GnRHR are expressed in DRG neurons

**Figure 1:**
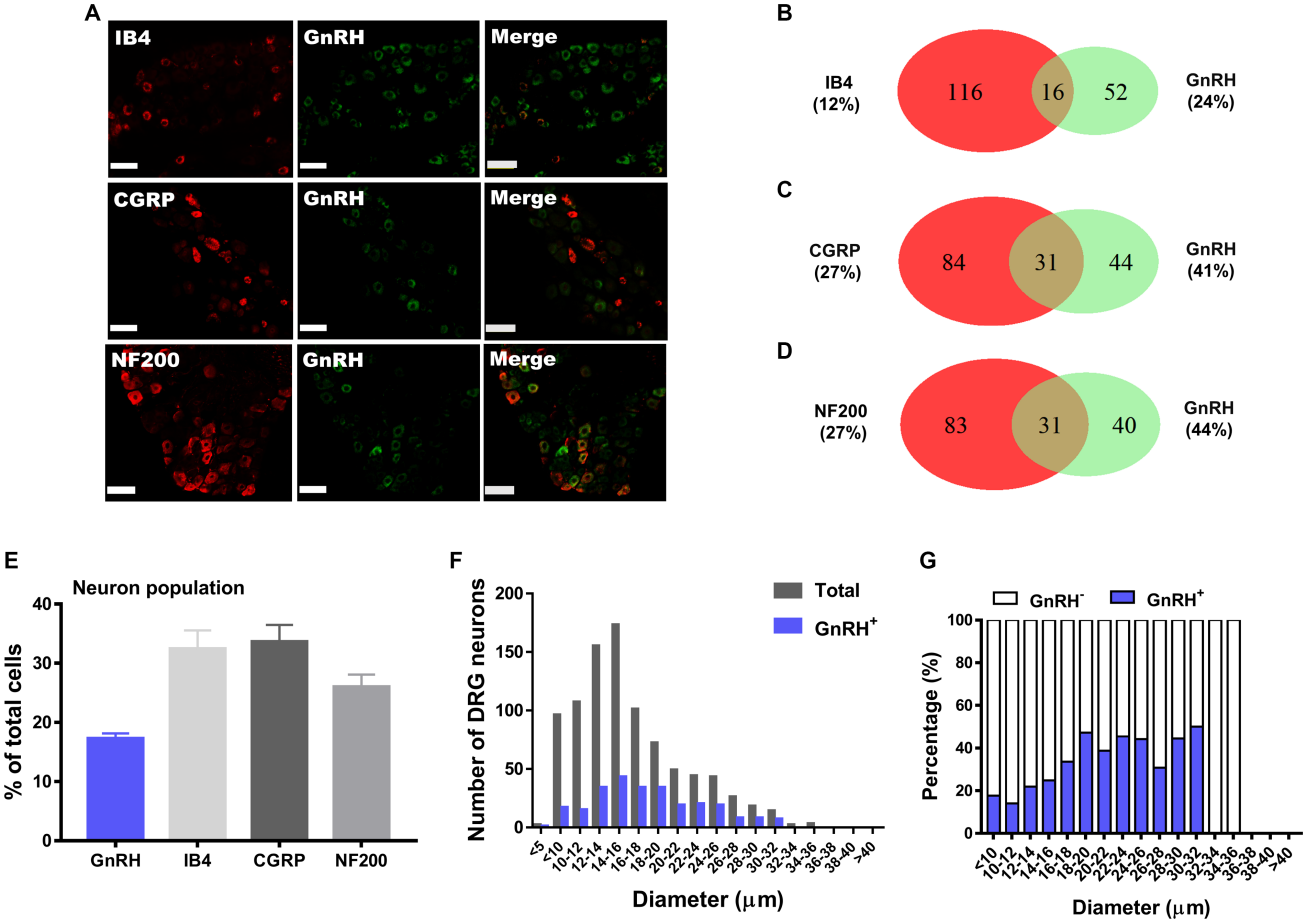
GnRH expression in a subset of DRG neurons. **(A)** Double immunostaining of GnRH with other DRG markers in the lumbar DRGs (scale bar, 50 μm). **(B–D)** Counts in the numbers of neurons with expression and co-expression of GnRH and other DRG markers and their ratios compared to the number of whole neuronal populations presented in Venn diagrams. **(E)** Expression ratios of GnRH-(+) and other DRG marker-(+) neurons presented in histograms as means ± S.E.M. **(F)** Size distribution of collected DRG neurons and GnRH-(+) DRG neurons according to soma diameters. **(G)** Percent distribution of GnRH-(+) neurons from **(F)** at each range of sizes.

**Figure 2:**
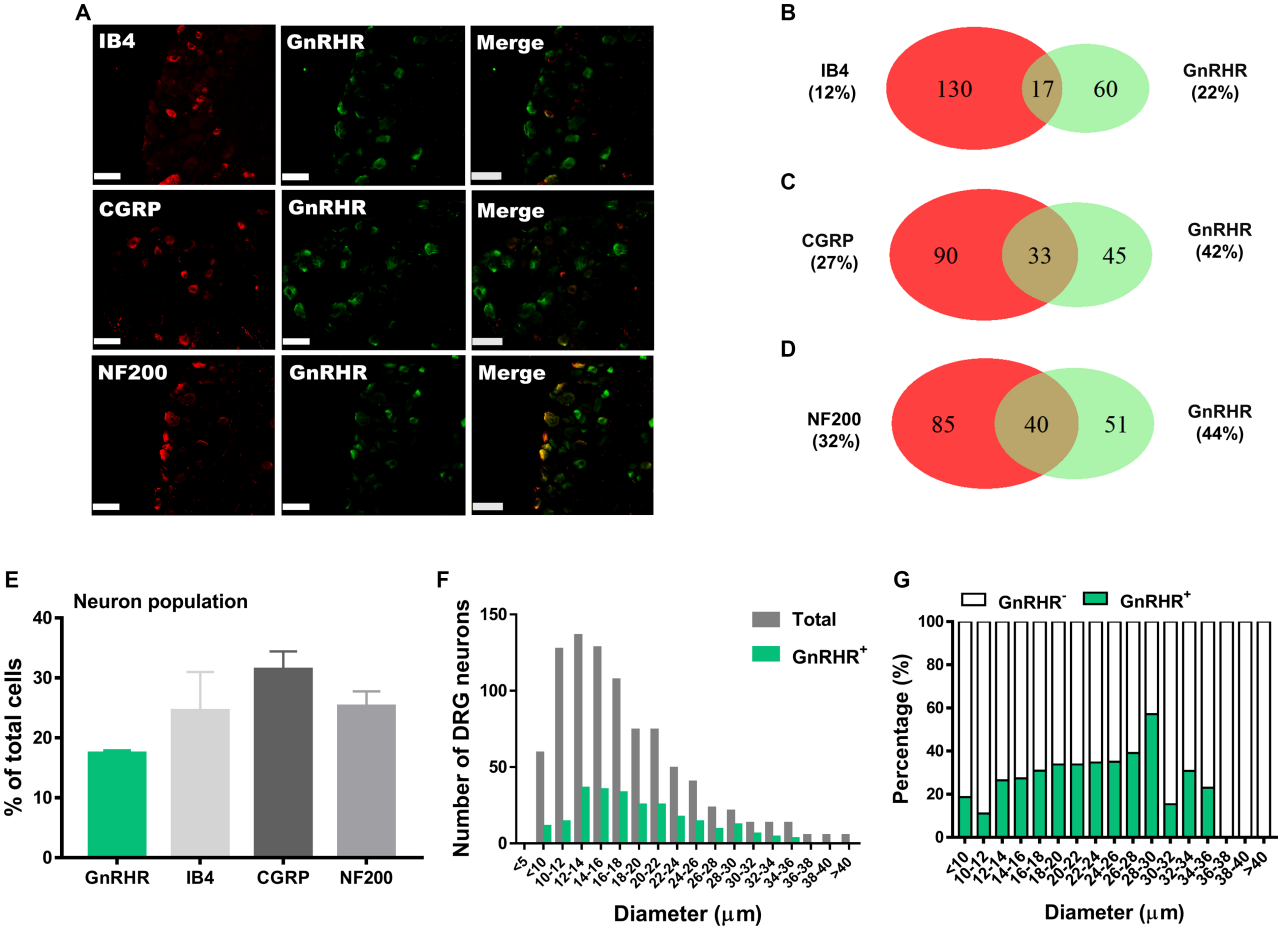
GnRHR expression in a subset of DRG neurons. **(A)** Double immunostaining of GnRHR with other DRG markers in the lumbar DRGs (scale bar, 50 μm). **(B–D)** Counts in the numbers of neurons with expression and co-expression of GnRHR and other DRG markers and their ratios compared to the number of whole neuronal populations presented in Venn diagrams. **(E)** Expression ratios of GnRHR-(+) and other DRG marker-(+) neurons presented in histograms as means ± S.E.M. **(F)** Size distribution of collected DRG neurons and GnRHR-(+) DRG neurons according to soma diameters. **(G)** Percent distribution of GnRHR-(+) neurons from **(F)** at each range of sizes.

We hypothesize that GnRH and GnRHR interaction may occur and contribute to peripheral nociception since we found that the frequencies of various pain-related adverse effects in patients when treated with GnRH analogues are noticeable according to our reconstruction of Sider 4.1 information^1^ (Supplementary Figure S1). First, to verify the presence of GnRH and GnRHR in somatosensory neurons, we investigated their expression in those neurons in separate datasets by analyzing massive RNA-seq profiles (Gene Expression Omnibus resource numbers: GSE59739 and GSE63576) from two recent independent studies on murine DRG neurons (Usoskin et al., 2015; Li et al., 2016). Uniform Manifold Approximation and Projection (UMAP)-based construction of their raw transcriptomic data identified a subset of GnRH and GnRHR expresser cells in approximately multiple subclusters (Supplementary Figures S2, S3). When we profiled co-expression with subcluster marker genes using reconstructed UMAP representations of GSE59739 (Supplementary Figure S2) and GSE63576 datasets (Supplementary Figure S3), Calca (encoding calcitonin-gene related peptide {CGRP}) and Nefh (encoding neurofilament 200 {NF200}) were overlapped, indicating that GnRH and GnRHR are expressed both in unmyelinated C-fiber nociceptors and myelinated A-fiber neurons. The expressions were reproducible by immunohistochemistry (Figures 1A,C–E, 2A,C–E). According to isolectin B4 (IB4) staining, part of non-peptidergic C-fiber neurons also express those ligand and receptor (Figures 1B, 2B). The GnRH and GnRHR were expressed mostly in small to medium diameter neurons, which typically represent C- and Aδ fiber nociceptors (Figures 1F,G, 2F,G). Consequently, we found GnRH and GnRHR-positive subclusters from the two independent RNA-seq datasets and confirmed their protein expressions experimentally. The result indicates their possible modulatory roles in pain mediation.

### 2.2 Peripheral GnRHR activation modifies mechanical pain

To examine whether peripheral GnRHR activation alters pain, we injected GnRH into local areas of experimental animals for stimulating somatosensory nerve terminals. No significant behavioral change was detected when GnRH was intraplantarly treated for monitoring nociceptive behaviors and neither was it detected when the ligand was applied to the nape of animals for monitoring pruritic behaviors which are known to be initiated by excitation of pruriceptor DRG neurons (Supplementary Figures S4A–C). We further checked whether GnRHR activation contributed to formalin-induced pain by observing the effect of antagonism using intraplantarly and intrathecal pretreatment of cetrorelix and no significant change was detected. The results suggest that GnRHR activation may not directly induce pain and pruritus.

**Figure 3:**
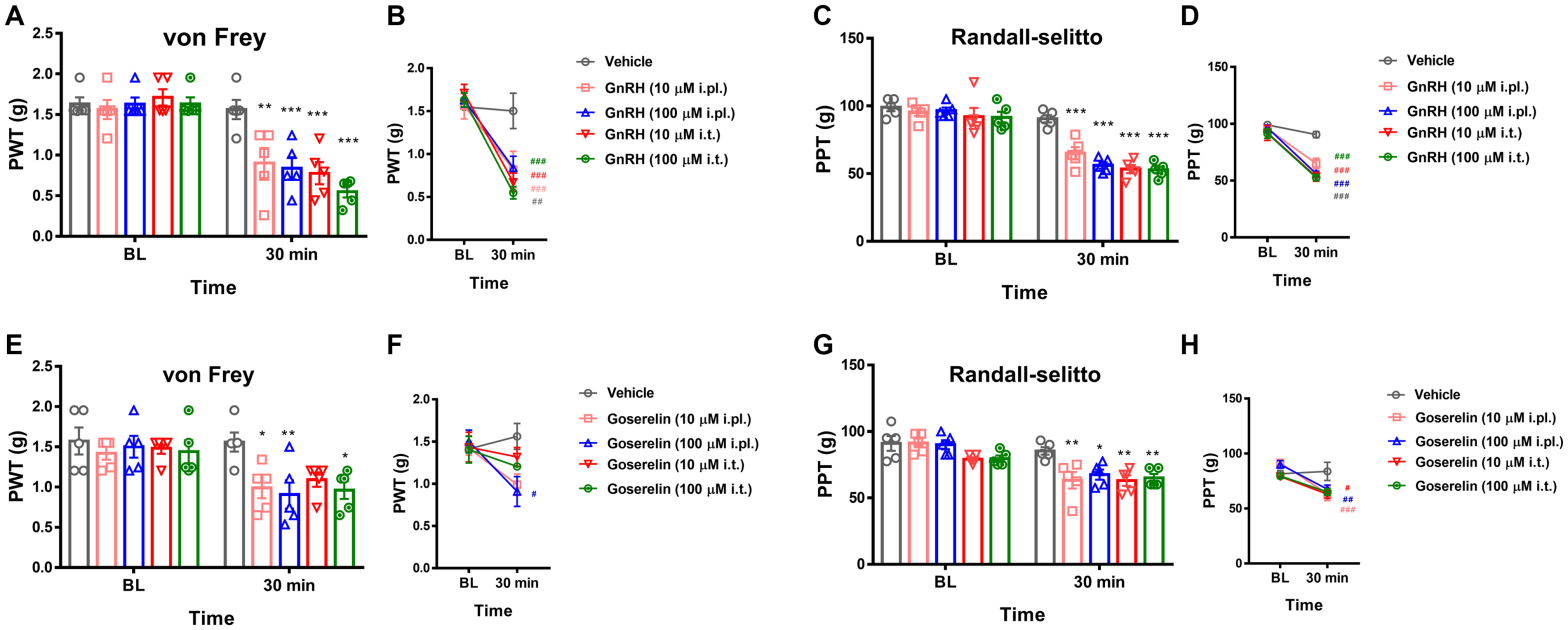
GnRHR activation sensitizes mechanical pain. **(A)** The paw withdrawal thresholds (PWT) upon the von Frey filament stimulations were obtained first for baseline (B.L.) averages and were measured again 30 min after drug treatment. Animals were intraplantarly (i.pl.) or intrathecally (i.t.) treated with vehicle (10 μL saline) or GnRH (10 μM or 100 μM). Statistic comparisons to the vehicle-treated group were performed. **(B)** Statistic comparisons to the averaged baseline of the same group before drug treatment were performed using the data shown in **(A)**. **(C)** The paw pressure thresholds (PPT) upon the Randall-Selitto stimulations were obtained first for baseline (B.L.) averages and were measured again 30 min after drug treatment. Animals were intraplantarly (i.pl.) or intrathecally (i.t.) treated with vehicle (10 μL saline) or GnRH (10 μM or 100 μM). Statistic comparisons to the vehicle-treated group were performed. **(D)** Statistic comparisons to the averaged baseline of the same group before drug treatment were performed using the data shown in **(C)**. **(E)** The paw withdrawal thresholds (PWT) upon the von Frey filament stimulations were obtained first for baseline (B.L.) averages and were measured again 30 min after drug treatment. Animals were intraplantarly (i.pl.) or intrathecally (i.t.) treated with vehicle (10 μL saline) or goserelin (10 μM or 100 μM). Statistic comparisons to the vehicle-treated group were performed. **(F)** Statistic comparisons to the averaged baseline of the same group before drug treatment were performed using the data shown in **(E)**. **(G)** The paw pressure thresholds (PPT) upon the Randall-Selitto stimulations were obtained first for baseline (B.L.) averages and were measured again 30 min after drug treatment. Animals were intraplantarly (i.pl.) or intrathecally (i.t.) treated with vehicle (10 μL saline) or goserelin (10 μM or 100 μM). Statistic comparisons to the vehicle-treated group were performed. (H) Statistic comparisons to the averaged baseline of the same group before drug treatment were performed using the data shown in **(G)**.

We then focused on possible modulatory roles of GnRHR activation in physical pain mediation. When we intraplantarly or intrathecally injected GnRH for stimulating the peripheral and central terminals of somatosensory neurons respectively, both treatments significantly lowered von Frey (stinging) and Randall-Selitto (pressure) thresholds, while Hargreaves (heat) threshold was relatively tolerant (Figures 3A–D and Supplementary Figures S4H–I). Such mechanical phenotype-specific effects were reproducible in tests with the administration of another GnRH analogue, goserelin (Figures 3E–H and Supplementary Figures S4J–K). In addition, the effects of GnRH occurred in a dose-dependent fashion (Supplementary Figures S4L–P). Collectively, peripheral GnRHR activation appears to not elicit but enhance mechanical pain.

We then asked whether modality-specific molecule-mediated acute pain phenotypes were also modified by GnRHR activation. Capsaicin-induced nociceptive behaviors, which reflect acute transient receptor potential vanilloid subtype 1 ion channel (TRPV1) activation of nociceptors, were enhanced only in a limited range of the monitoring period (Supplementary Figure S4Q). Cinnamaldehyde-induced behaviors, which reflect acute ankyrin subtype 1 TRP ion channel (TRPA1) activation of nociceptors, were enhanced to a greater extent (Supplementary Figure S7R). On the other hand, KCl-induced behaviors, which reflect neuronal depolarization caused by the activation of voltage-gated channels of nociceptors, were not altered (Supplementary Figure S7S). Collectively, TRPA1 may be a component most strongly affected by GnRHR activation in the modality test sets, and because TRPA1 contributes to nociceptive mechanotransduction, the results seem to accord with those derived from the physical stimulation-based approaches above.

### 2.3 GnRHR activation modulates DRG neuronal functions

**Figure 4:**
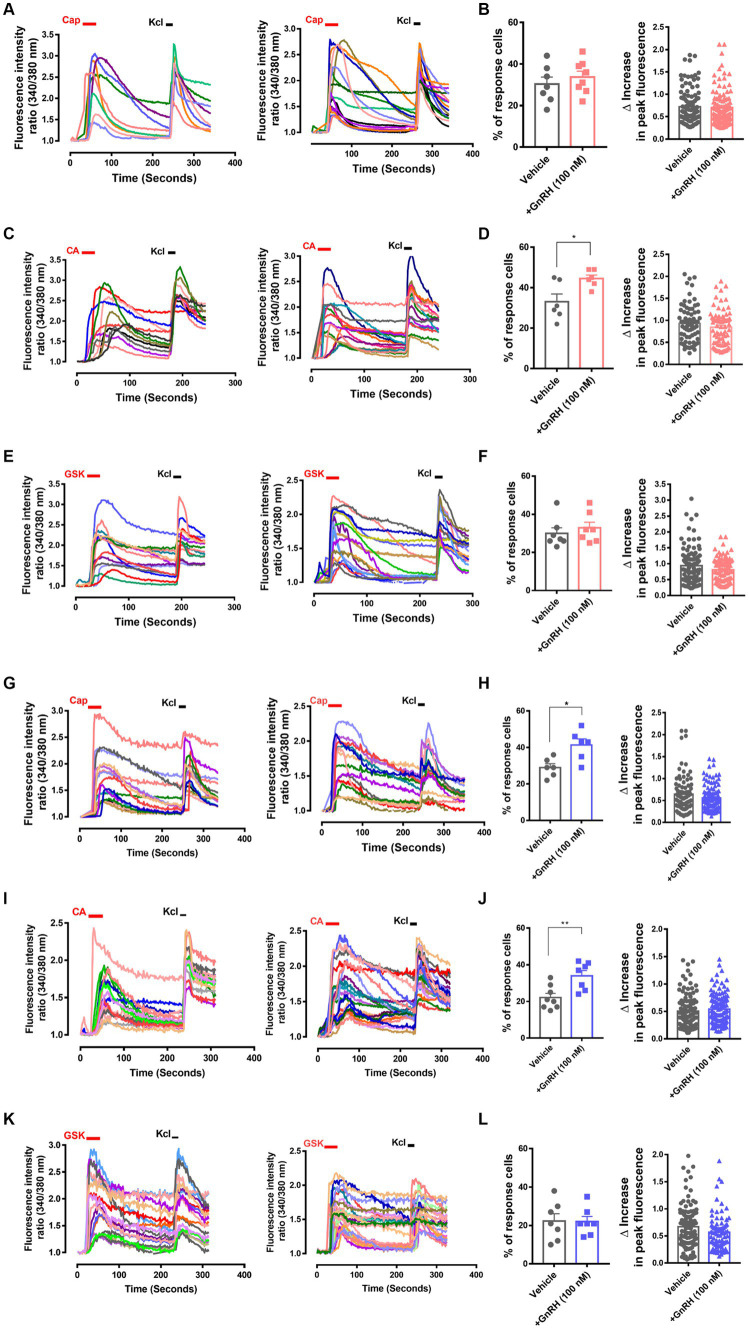
GnRH, when 30 min or 24 h incubated, modulates TRPA1 and TRPV1 functions in DRG neurons. **(A)** Representative traces for intracellular Ca^2+^ increases by 0.3 μM capsaicin (CAP)-induced TRPV1 activation and by 60 mM KCl-induced depolarization in Fura-2 Ca^2+^ imaging experiments using cultured murine DRG neurons with (right) or without 30 min pre-incubation of 100 nM GnRH (left). **(B)** Summary of % CAP-responder neurons among KCl-responder neurons at each culture batch (left) and collection of the amplitudes of highest Ca^2+^ peaks per each neuron (right) in **(A)**. **(C)** Representative traces for intracellular Ca^2+^ increases by 300 μM cinnamaldehyde (CA)-induced TRPA1 activation and by 60 mM KCl-induced depolarization in Fura-2 Ca^2+^ imaging experiments using cultured murine DRG neurons with (right) or without 30 min pre-incubation of 100 nM GnRH (left). **(D)** Summary of % CA-responder neurons among KCl-responder neurons at each culture batch (left) and collection of the amplitudes of highest Ca^2+^ peaks per each neuron (right) in **(C)**. **(E)** Representative traces for intracellular Ca^2+^ increases by 10 nM GSK1016790A (GSK)-induced TRPV4 activation and by 60 mM KCl-induced depolarization in Fura-2 Ca^2+^ imaging experiments using cultured murine DRG neurons with (right) or without 30 min pre-incubation of 100 nM GnRH (left). **(F)** Summary of % GSK-responder neurons among KCl-responder neurons at each culture batch (left) and collection of the amplitudes of highest Ca^2+^ peaks per each neuron (right) in **(E)**. **(G)** Representative traces for intracellular Ca^2+^ increases by 0.3 μM capsaicin (CAP)-induced TRPV1 activation and by 60 mM KCl-induced depolarization in Fura-2 Ca^2+^ imaging experiments using cultured murine DRG neurons with (right) or without 24 h pre-incubation of 100 nM GnRH (left). **(H)** Summary of % CAP-responder neurons among KCl-responder neurons at each culture batch (left) and collection of the amplitudes of highest Ca^2+^ peaks per each neuron (right) in **(G)**. **(I)** Representative traces for intracellular Ca^2+^ increases by 300 μM cinnamaldehyde (CA)-induced TRPA1 activation and by 60 mM KCl-induced depolarization in Fura-2 Ca^2+^ imaging experiments using cultured murine DRG neurons with (right) or without 24 h pre-incubation of 100 nM GnRH (left). **(J)** Summary of % CA-responder neurons among KCl-responder neurons at each culture batch (left) and collection of the amplitudes of highest Ca^2+^ peaks per each neuron (right) in **(I)**. **(K)** Representative traces for intracellular Ca^2+^ increases by 10 nM GSK1016790A (GSK)-induced TRPV4 activation and by 60 mM KCl-induced depolarization in Fura-2 Ca^2+^ imaging experiments using cultured murine DRG neurons with (right) or without 24 h pre-incubation of 100 nM GnRH (left). **(L)** Summary of % GSK-responder neurons among KCl-responder neurons at each culture batch (left) and collection of the amplitudes of highest Ca^2+^ peaks per each neuron (right) in **(K)**. All experiments were triplicated and each column represents the mean ± S.E.M. (^*^p < 0.05, ^**^p < 0.01, unpaired two-tailed Student’s *T*-test).

To interrogate whether DRG neuronal functions could explain those nociceptive modulations, we analyzed GnRH action in intracellular fluorescent Ca^2+^ imaging with cultured DRG neurons (Figures 4A–L). Although the amplitude of the neuronal Ca^2+^ peaks was unaffected, the responder population upon cinnamaldehyde treatment, indicating the number of TRPA1-(+) neurons, was increased by 30-min GnRH incubation (Figures 4C,D). On the other hand, neither the amplitude nor the population for TRPV1-mediated and capsaicin-prompted Ca^2+^ increases was altered upon GnRH incubation. With a longer incubation of 24 h, both the numbers of TRPA1- and TRPV1-mediated responders were increased and changes in the TRPA1-(+) population were statistically more significant (Figures 4G–J). In contrast, TRPV4-mediated responder neurons, which were monitored using GSK1016790A applications, were largely inert to GnRH treatment (Figures 4E,F,K–L). Ca^2+^ increases by KCl-induced depolarization also showed no shift when GnRH treated for either shorter or longer periods of time (Supplementary Figures S5A–E). Therefore, GnRH may contribute to population growth with TRPA1 and to a lesser extent, with TRPV1 in DRG sensory neurons, appearing to somewhat correspond to our data at *in vivo* aspects above. Because protein expression of TRPV1 and TRPA1 in these neurons was tolerant to GnRH activation and inhibition, the population growth may depend on other mechanisms, including post-transcriptional modulations (Supplementary Figures S5F–H) (Takahashi and Mori, 2011; Utreras et al., 2012; Rowan et al., 2014; Voolstra and Huber, 2014). In a small subset of neurons, GnRH led to a marginal increase in Ca^2+^ fluorescence or perturbation of TRPA1 responses, which might be an interesting action but were not statistically significant (Supplementary Figure S6).

### 2.4 Peripheral GnRHR activation is involved in pathologic mechanical pain

**Figure 5:**
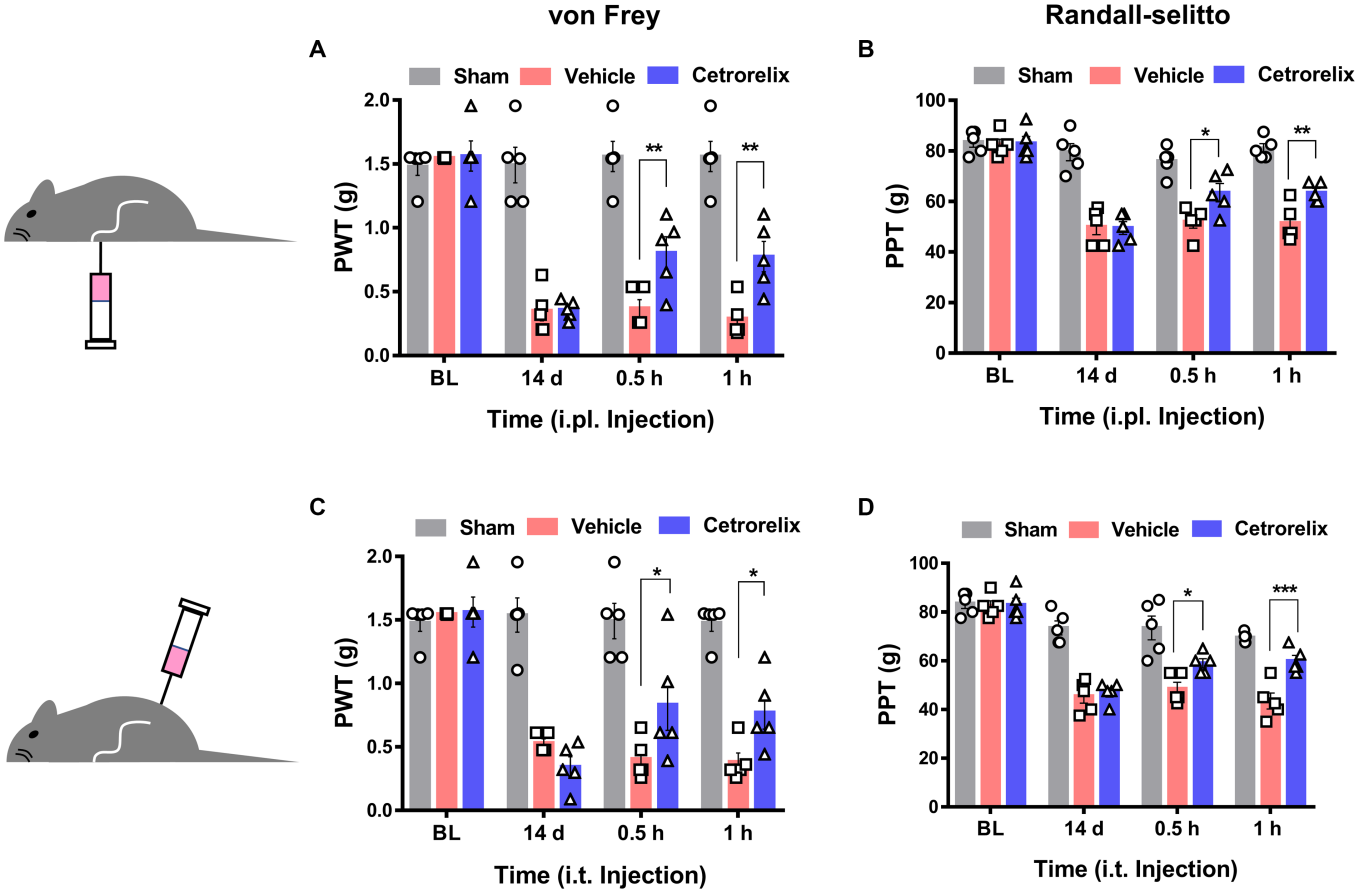
GnRH contributes to neuropathic pain. **(A)** Time course of von Frey thresholds in mice with chronic constriction injury (CCI). Animals were intraplantarly treated with vehicle (pink) or cetrorelix (10 μM, blue) in the ipsilateral hind paws 14 days after CCI surgery. **(B)** Time course of Randall-Selitto thresholds in mice with CCI. Animals were intraplantarly treated with vehicle (pink) or cetrorelix (10 μM, blue) in the ipsilateral hind paws 14 days after CCI surgery. **(C)** Time course of von Frey thresholds in mice with CCI. Animals were intrathecally treated with vehicle (pink) or cetrorelix (10 μM, blue) 14 days after CCI surgery. **(D)** Time course of Randall-Selitto thresholds in mice with CCI. Animals were intrathecally treated with vehicle (pink) or cetrorelix (10 μM, blue) 14 days after CCI surgery.

Based on the sensory neuron-mediated outcomes described above, we further hypothesized that GnRH may work peripherally in pathologic states of pain. When we first tested GnRH antagonism using a model of complete Freund’s adjuvant (CFA)-induced hind paw inflammation, hyperalgesic inflammatory pain responses were not significantly changed upon ipsilateral intraplantarly or intrathecal injections of cetrorelix (Supplementary Figures S7A–D). On the other hand, the same antagonism was effective in alleviating chronic constriction injury (CCI)-induced pain, indicating that endogenous GnRH play a role in neuropathic states. In contrast to those treated with vehicle, mice under CCI condition experienced reduced mechanical allodynia and hyperalgesia when treated with cetrorelix intraplantarly (Figures 5A,B) and intrathecally (Figures 5C,D), in a statistically significant manner. Since TRPA1 responses were shifted in DRG neurons above and this channel is known as a mechanical and cold sensor (Bang and Hwang, 2009), it may not only contribute to this altered mechanical phenotype but also to a cold one and we tested whether cold allodynia is also affected. In the same manner, cold allodynia was alleviated by cetrorelix treatment only in CCI conditions (Supplementary Figures S7E–H).

**Figure 6:**
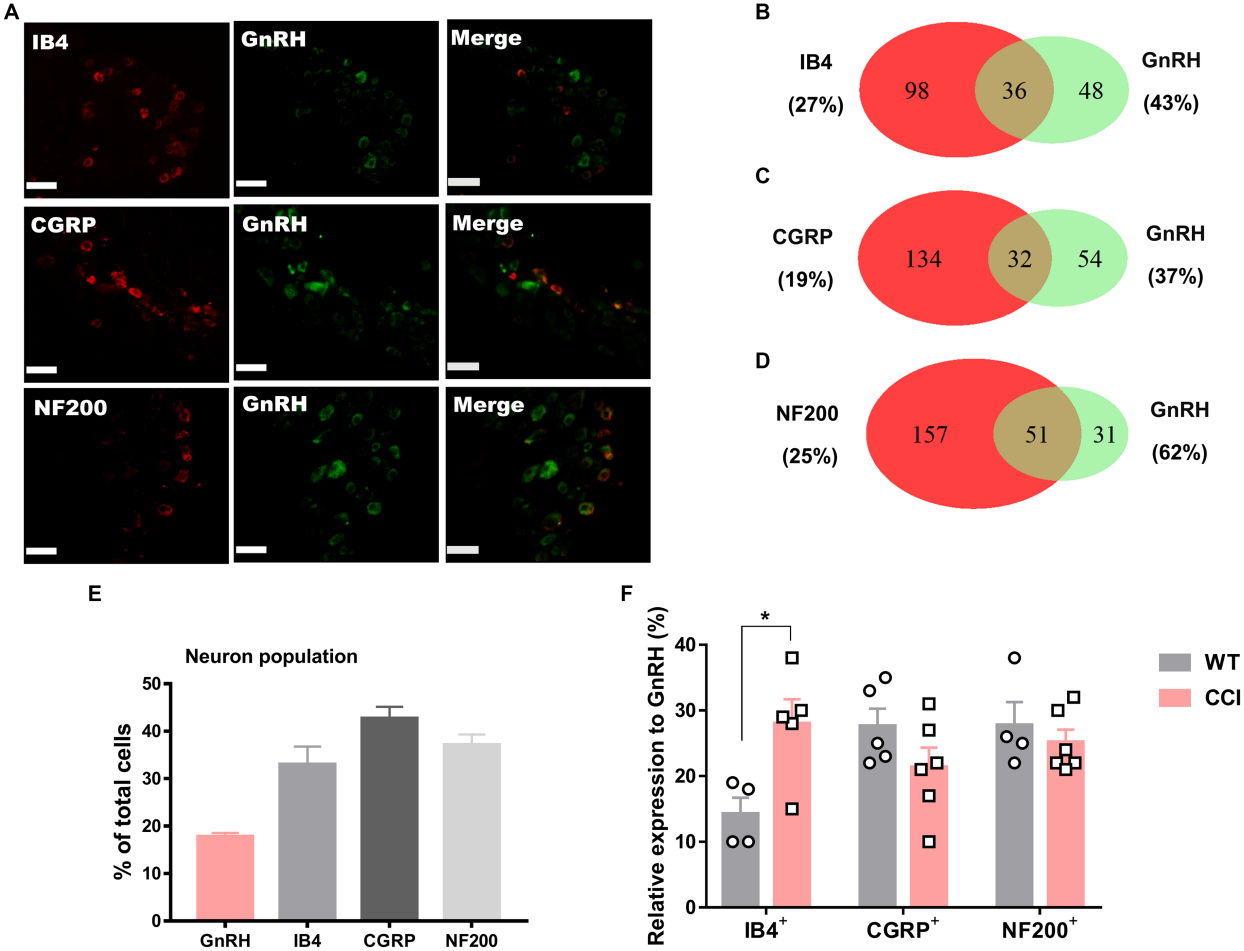
GnRH expression in a subset of DRG neurons under CCI condition. **(A)** Double immunostaining of GnRH with other DRG markers in the lumbar DRGs under CCI condition (scale bar, 50 μm). **(B–D)** Counts in the numbers of neurons with expression and co-expression of GnRH and other DRG markers and their ratios compared to the number of whole neuronal populations presented in Venn diagrams. **(E)** Expression ratios of GnRH-(+) and other DRG marker-(+) neurons presented in histograms as means ± S.E.M. **(F)** Expression ratios of GnRH-(+) among other DRG marker-(+) neurons and their comparison to those from Figures 1B–D.

**Figure 7:**
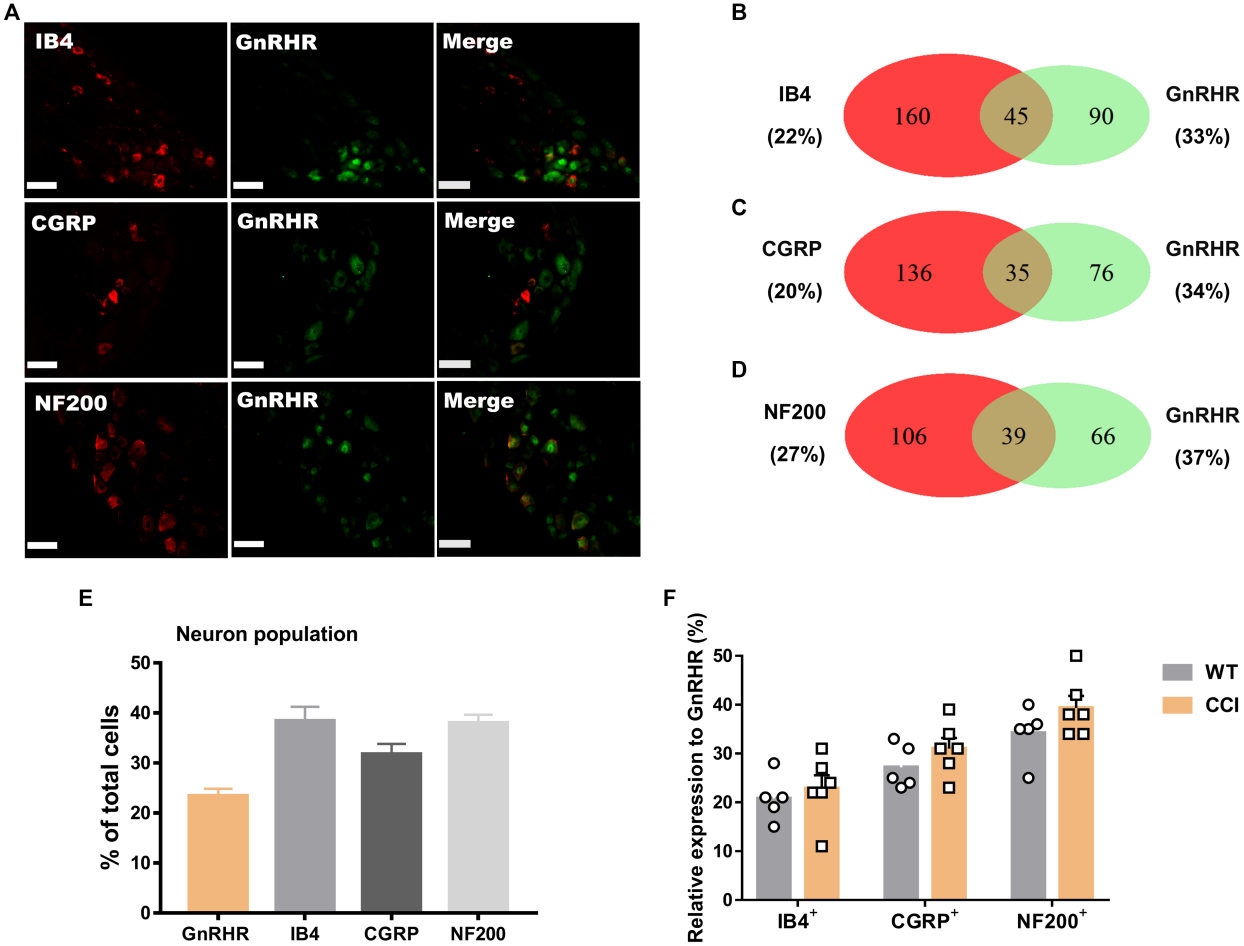
GnRHR expression in a subset of DRG neurons under CCI condition. **(A)** Double immunostaining of GnRHR with other DRG markers in the lumbar DRGs under CCI conditions (scale bar, 50 μm). **(B–D)** Counts in the numbers of neurons with expression and co-expression of GnRHR and other DRG markers and their ratios compared to the number of whole neuronal populations presented in Venn diagrams. **(E)** Expression ratios of GnRHR-(+) and other DRG marker-(+) neurons presented in histograms as means ± S.E.M. **(F)** Expression ratios of GnRHR-(+) among other DRG marker-(+) neurons and their comparison to those from Figures 2B–D.

We wondered whether GnRH could cause pain in the process of contributing as a trophic player because it has been shown to exhibit a neurotrophic effect in the central nervous system (CNS). Observations on nerve morphology and sensory functions in a sciatic nerve crush model indicated that, both the morphological and sensory functional restorations were mildly improved by extraneous GnRH treatment (Supplementary Figures S8A–F). These results suggest that, rather than under inflammation, roles of GnRH in pain exacerbation may be more obvious under neuropathic conditions, and that possibly because of the presence of local GnRH that may contribute to restoring the neuropathy itself. However, the effect on expression was somewhat limited when we quantitated GnRH-(+) (Figures 6A–F) and GnRHR-(+) (Figures 7A–F) neurons in our immunostaining using normal mice and mice under CCI condition. Only the expression of GnRH-(+) subpopulation in IB4-(+) DRG neurons was significantly increased (Figure 6F).

We showed that GnRHR activation induces no acute pruritic symptoms (Supplementary Figure S4). Additionally, we wondered GnRH begins to participate in pathologic pruritus under a disease state. In an allergic contact dermatitis model using 1-chloro-2,4-dinitrobenzene (DNCB), which is also known to partly reflect an atopic condition, GnRH antagonism did not significantly alter the scratching phases (Supplementary Figure S8G). Interestingly, GnRH agonism slightly aggravates those behaviors at a few time points, accompanied by worsened skin pathology, probably owing to the vicious cycle of itch-scratch (Supplementary Figures S8H–J). The result suggests that endogenous GnRH-GnRHR interactions in pruritic pathology were less predictable, but the intrinsic presence of GnRHR itself may be negatively considered in a situation where GnRH treatment is intended.

Overall, our major findings are that GnRH and GnRHR interactions, despite possibly being beneficial in a neurotrophic aspect in the periphery, may exacerbate mechanical pain particularly in neuropathic conditions. Therefore, this study suggests a new modulatory mechanism in peripheral sensory neurons based on a neuropeptide that is known to be active mainly in the CNS, also contributing to the mechanistic understanding of practical situations in GnRH-related drug treatment.

## 3 Discussion

In this study, we demonstrated that GnRH, a central peptide hormone, can peripherally modulate nociception via receptor-dependent mechanisms in DRG neurons. A number of known peptide hormones in the CNS have been revisited recently as a peripherally acting neuropeptides, particularly on DRG neurons of which subpopulations express their own GPCRs such as corticotropin-releasing hormone receptors, somatostatin receptors, oxytocin receptors, GPR171, etc., (Zheng et al., 2020; Cho et al., 2021; Zheng et al., 2021). Such versatile expression in peripheral neurons may possibly be due to the essential role of widely monitoring changes in the internal environment. A possible vigilant role that includes an alert for brain damage, for example, by evaluating unusually increased levels of brain-specific constituents such as GnRH and other hormones, may require thorough investigation (Edvinsson et al., 2019). Another possibility can be the need to tune their ascending signals to an appropriate range, to protect the body from external signals that can often deviate from an internal homeostatic capacity. Circuit-based opioid modulation and the local resolution of the neuroinflammatory phase by resolvin and maresin receptors may represent such cases (Yoo et al., 2013; Lim et al., 2015; Bagley and Ingram, 2020). At a different viewpoint, owing to the physical distance from the brain, peripheral ganglionic neurons may have been under evolutionary pressure to develop a differential survival strategy to utilize local resources for autocrine and/or paracrine health signals. In some cancer types, for example, the GnRH autocrine system regulates cell proliferation (Grþndker and Emons, 2017). Receptor-mediated GnRH actions in the periphery explored in this study are likely based on these combined reasons. The latter ones seem to be at least roughly predictable in our experimental setting in the current stage where few reports are available regarding GnRH-(+) descending modulatory circuit.

Previously, GnRHR activation by its ligand was shown to promote the excitability of target cells by modulating voltage-dependent channels such as Na^+^ and K^+^ channels, probably contributing to next-step secretions on the hormonal axis or cell fate modification (Maiti et al., 2005; Zhang and Delay, 2007; Hirdes et al., 2010). In contrast, our surrogate measurement of those depolarization effects using KCl-induced Ca^2+^ influx, which reflects the operations of whole DRG neuronal voltage-dependent components forming action potentials, did not detect significant changes. Interestingly, DRG-enriched sensory TRP channel activities were statistically affected by GnRHR activation in our experiments. Among these TRPs, changes in TRPA1-mediated responses were most prominent. TRPA1 is known to participate in the sensing of nociceptive mechanical stretches, which may at least partly explain the specificity of the GnRH effect *in vivo* on the shift of the mechanical threshold although there may be other unknown mechanotransducer molecules, which remains to be explored regarding the GnRH effect (Kwan et al., 2006; Petrus et al., 2007; Brierley et al., 2011; Nassini et al., 2011). Altered cold sensation in our supplementary results may additionally support this TRPA1 involvement because TRPA1 is also a cold sensor (Bandell et al., 2004). Despite the number of capsaicin-responder neurons being moderately increased upon GnRH treatment in the analyses *in vitro*, heat-induced behaviors *in vivo* were largely unaffected. A limited population of GnRHR expressers may be insufficient to cover the whole heat-responsive nociceptors and shift the phenotype. Otherwise, because TRPV1 has also been shown to act as a mechanotransducer molecule, mechanical phenotypes were more vulnerable when the effects were assumed to be additive to TRPA1-mediated mechanotransduction (Birder et al., 2002). Another possibility is that GnRHR signal transduction may affect Piezo2 contribution in relatively large-diameter Aβ fibers in allodynic standpoint but, how the Piezo2-positive fibers are reorganized during the spinal circuit remodeling, particularly in neuropathic situations, remains to be further explored (Moehring et al., 2018).

Receptor-mediated metabotropic signals are considered to exquisitely operate for regulating TRP channel activity in sensory neurons. For example, downstream phosphorylation and dephosphorylation of channel proteins by kinases and phosphatases frequently tune channel activities (Bhave et al., 2002; Jung et al., 2004; Moriyama et al., 2005). In addition, mobilization and/or *de novo* generation of intracellular endogenous molecules during signaling cascades may directly bind and modulate TRPs, too (Shin et al., 2002; Watanabe et al., 2003; Dai et al., 2007). Altered channel expression or trafficking is another important mechanism for adjusting their responses at cellular levels (Ji et al., 2002; Zhang et al., 2005; Stein et al., 2006; Akopian et al., 2007; Camprubï-Robles et al., 2009; Schmidt et al., 2009; Ortïz-Renterïa et al., 2018). GnRHR-mediated intracellular enzymatic cascade closely shares many kinase enzymes such as protein kinase C and mitogen-activated protein kinases with those promoting surface translocation of TRPA1 and TRPV1 proteins, which could explain the GnRH-facilitated increase in TRP responders among DRG neurons found in this study (Cheung and Wong, 2008; Naor, 2009). Discrepancies were that changes in response amplitudes were not obvious whereas responder populations were clearly enlarged. It is also noteworthy that such increases took a relatively long GnRH exposure and that the magnitude of their effectiveness differentially depended on specific TRP channels. Further clarification is needed into whether a tighter subcellular coupling between downstream enzymes and particular effector channels for signal transduction occurs while there is a relatively less efficient interaction for some GnRHR-induced components.

Among our pathologic models, the GnRHR blockade was only effective at blunting neuropathic pain but not inflammatory pain including CFA-induced pain and formalin-induced 2nd phase responses. These results may suggest that local endogenous GnRH actions were only noticeable in neuropathic conditions. Indeed, both morphologically and functionally, somatosensory restorations were enhanced by GnRH treatment, indicating that GnRHR activation may contribute to peripheral nerve recovery. In addition, increases in the GnRHR expresser population were significant in IB4-(+) non-peptidergic neurons, which suggests that at least a portion of neurons switch to using the signals more actively to promote their restoration by altering receptor expression. Despite requiring further thorough developmental approaches as in the CNS, this can be another novel role of GnRH selectively defined in the peripheral nervous system, and perhaps pain exacerbation is a concomitant phenotype in fulfilling this role, reminiscing of the nerve growth factor, which is also a major contributor to pain generation (Mizumura and Murase, 2015; Martïnez-Moreno et al., 2018). Otherwise, however, considering its quantitatively mild shift of the restoration, it may be among positive outcomes often detected in Gq-coupled signal promotion in growth regulation.

By sorting the prevalent adverse effects of clinically available GnRH analogues, here we encountered the unexpected peripheral roles of GnRH and its receptor in pain centered in primary afferent neurons. This study suggests that the promotion of TRP channel-based mechanical nociception may be among important mechanisms in GnRH-mediated pain exacerbation. For pain management, this new information may refer to various therapeutic fields where GnRH agonists are used clinically. In addition, it may further contribute to enriching our perspectives regarding the modulatory concepts for peripheral somatosensory physiology by the neuropeptides that are known primarily as central hormones.

## 4 Materials and methods

### 4.1 Behavioral tests

The experiments were carried out under the approval of the institutional animal care and use committee of the university. Six-week-old male C57BL/6 J mice were used. Time engaged in hind paw licking and flicking behavior was quantitated for 5 min as described previously (Bandell et al., 2004; Bang et al., 2011; Choi et al., 2019). To elicit TRPV1 activation-induced behaviors, 100 ng capsaicin-containing 10 μL saline was intraplantarly injected. For eliciting TRPA1 activation-induced behaviors, 26.4 μg cinnamaldehyde in 20 μL saline was injected. For eliciting depolarization-induced behaviors mediated by voltage-gated channel activation, 140 mM KCl in 10 μL water was injected. To observe whether GnRH and its analogues induced any behaviors, 10 μM GnRH or 10 μM goserelin in 10 μL were injected in the same manner. *To observe formalin*-induced acute and tonic pain, 5% formalin in saline (20 μL) was intraplantarly injected and the cumulative time that a mouse spent licking and flinching the injected paw was measured every 5 min for 45 min. Hargreaves assay (using Plantar Analgesia Meter; IITC, Woodland Hills, CA, United States) for acute heat avoidance or heat hyperalgesia, von Frey assay by up-and-down paradigm (using von Frey filaments; Stoelting, Dale Wood, IL, USA) for mechanical allodynia, and Randall–Selitto assay (using Analgesy-Meter, UGO Basile, Italy) for flexion reflex to noxious pressures, were carried out as described previously (Moqrich et al., 2005; Kim et al., 2009; Bang et al., 2012). Acetone evaporation test for cold responses was performed as described previously (Kim et al., 2023a).

For pruritic behavior measurements, the following compounds were injected into the nape of mice: 500 μg/50 μL histamine, 200 μg /50 μL chloroquine in saline as described previously (Kim et al., 2023b). To observe whether GnRH and its analogues induced any behavior, 10 μM GnRH or 10 μM goserelin in 50 μL were injected in the same manner.

### 4.2 Pathologic pain and pruritus models

For CFA-induced inflammation, 10 μL of CFA was injected into a hind paw. In order to determine changes in mechanical or thermal behaviors over 24 h and 48 h CFA inflammation, mice were acclimated to the test environment for 30 min before performing the following assays.

For neuropathic pain development, the CCI model was established as described previously (Bennett and Xie, 1988). Briefly, the mice were anesthetized with inhalation of 3% isoflurane in a mixture of N_2_O/O_2_ gas. The full circumference of the sciatic nerve of the left hind limb was exposed and loosely tied three times with two silk sutures (7–0; Ailee, Busan, Korea). Sham surgery was conducted by exposing the sciatic nerve in the same manner, but without tying the nerve. Hargreaves and von Frey assays were performed with the injured mice 14 days after surgery.

For modeling nerve crushes, mice received a single unilateral crush injury to the sciatic nerve (Davies et al., 2019). Briefly, under isoflurane anesthesia as described above, the left thigh was shaved, iodine was spread, and an incision was made in the mid-thigh. The sciatic nerve was exposed and carefully freed of connective tissues and crushed for 15 s using fine, mirror finished forceps (11–412-12, KASCO, Pakistan). The wound was then sutured, and the mice received GnRH intrathecally on 3 consecutive days. Following the injury, mechanical sensitivity was determined using von Frey filaments with a 2 g bending force. Mice were tested once every 2 days after the nerve was crushed. For the sham-operated controls, the sciatic nerve was exposed but not injured.

For the allergic contact dermatitis tests, a DNCB 1% dissolved in 150 μL acetone: olive mixture (3:1 vol/vol) was spread on the shaved dorsal skin of 5-week-old male BALB/C mice once every day for 3 days. Once dermatitis was induced, 0.5% DNCB was spread to maintain the condition once every 2 days for 2 weeks. Meantime, vehicle (50 μL/day), cetrorelix or GnRH was intradermally administered. Dorsal skin was sampled from mice that were anesthetized and sacrificed at the end of the treatment After hematoxylin and eosin (H&E) staining, 6-μm thick skin sections from the dorsal skin was observed microscopically (BX51-P, Olympus) and analyzed using ImageJ.

### 4.3 Immunohistochemical analysis

Lumbar DRGs from mice were fixed in 4% paraformaldehyde for 4 h, and cryoprotected overnight at 4°C in 30% sucrose. Then 14-μm sections were prepared using a Leica CM3050s cryotome (Leica Microsystems, Wetzlar, Germany) and were permeabilized with 0.2% Triton-X100 for 1 h and then stained with anti-GnRHR (rabbit, 1:1000; Abcam), anti-GnRH (rabbit, 1:1000, Invitrogen), anti-cGRP (goat, 1:2000; Abcam), and anti-NF200 (mouse, 1:100; Sigma-Aldrich) antibodies. After 24 h at 4°C, sections were stained with secondary antibodies (Alexa 594-labeled or Alexa 488-labeled secondary antibodies, and Alexa 594-conjugated IB4, Life Technologies) for 1 h at room temperatures and placed under a coverslip, followed by image acquisition with an iRiS Digital imaging system (Logos Biosystems, Anyang, Korea) and/or Zeiss LSM 800 confocal microscope (Carl Zeiss, Oberkochen, Germany).

### 4.4 Neuron cultures and fluorescence intracellular Ca^2+^ imaging experiments

Primary cultures of DRG neurons were prepared according to the protocol that we previously reported (Chiu et al., 2013). All neurons were grown at 37°C with 5% CO_2_ and were used at 48–72 h after culture. The Ca^2+^ imaging experiments were conducted as described previously (Chatzigeorgiou et al., 2013). Briefly, neurons were loaded with 5 μM Fura-2 acetoxymethyl dye and 0.02% pluronic F127 for 30 min. The neurons were resuspended in 140 mM NaCl, 5 mM KCl, 2 mM CaCl_2_, 1 mM MgCl_2_, 10 mM Glucose, and 10 mM HEPES (titrated to pH 7.4 with NaOH). Images of dye-loaded neurons were obtained with a cooled CCD camera (Retiga-SRV, Q-imaging Corp., Burnaby, BC, Canada). The ratio of fluorescence intensity at 340 nm/380 nm wavelengths in each experiment was analyzed using MetaFluor (Molecular Devices, Sunnyvale, CA, United States).

### 4.5 Single cell RNA-sequencing (scRNA-seq) data analysis

scRNA-seq datasets from Gene Expression Omnibus (GEO) accession numbers GSE59739 and GSE63576, respectively, were used as raw data. Gene count matrix log-normalization (10,000 scale factor), gene clustering, dimension reduction analysis (universal manifold approximation and projection; UMAP), differential gene expression analysis, and plotting were conducted using R (version 4.1.2) and Seurat (version 4.0.6). The data was imputed for denoising with Markov affinity-based graph imputation of cells (MAGIC, *t* = 8).

### 4.6 Western blots

Proteins in cells were extracted using RIPA buffer (R0278, Sigma-Aldrich, St. Louis, MO, United States). The cell lysate was centrifuged at 15,000 g for 10 min and was used for this assay using 10% sodium dodecyl sulfate–polyacrylamide gel electrophoresis (SDS-PAGE; Bio-Rad, Hercules, CA, United States). Transferring the protein from the gel to the Immobilon®-P PVDF Membrane (IPVH00010, Millipore Corp, Bedford, MA, United States). The transferred membranes were probed with polyclonal anti-TRPA1 (sheep, 1:200; Antibodies-online), monoclonal anti-TRPV1 (mouse, 1:100; Abcam), and monoclonal anti-GAPDH (1:10000, #2118, Cell Signaling Technology) antibodies, followed by horseradish peroxidase-conjugated goat anti-rabbit antibodies (1:10000, G-21234, Invitrogen, Carlsbad, CA, USA) before visualizing the proteins by Chemi-doc iBright 1000CL (ThermoFisher Scientific, Cleveland, OH, United States).

### 4.7 Compounds

All chemicals were purchased from Sigma-Aldrich (St. Louis, MO, United States) unless otherwise described. GnRH was purchased from Anygen (Gwangju, Korea). Cetrorelix and goserelin were purchased from Cayman Chemicals (Ann Arbor, MI, United States). Stock solutions were prepared using water or dimethyl sulfoxide and diluted with test solutions before use.

### 4.8 Data analysis

Statistical significance of data was assessed using the two-tailed unpaired Student’s *t*-test, one-way analysis of variance (ANOVA) followed by Tukey’s *post hoc* test, or two-way ANOVA followed by Tukey’s *post hoc* test (^****^p < 0.0001, ^***^p < 0.001, ^**^p < 0.01, ^*^p < 0.05). Data are shown as means ± S.E.M.

## Data availability statement

The datasets presented in this study can be found in online repositories. The names of the repository/repositories and accession number(s) can be found in the article/Supplementary material.

## Ethics statement

The animal study was approved by the institutional animal care and use committee of Korea University. The study was conducted in accordance with the local legislation and institutional requirements.

## Author contributions

HZ and SH: conceptualization and writing-original draft. HZ, MK, CK, JC, and SH: data curation. HZ, MK, YK, PC, HL, and SH: formal analysis. HZ, MK, CK, YK, PC, JL, HL, and H-IY: investigation. HZ, MK, CK, YK, PC, JL, HL, H-IY, and JC: methodology. HZ and MK: validation. MK and SH: writing-review & editing. PC, JL, H-IY, and SH: resources. JL, JC, and SH: project administration. JC and SH: supervision. SH: funding acquisition. All authors contributed to the article and approved the submitted version.

## Footnotes

^1^ http://sideeffects.embl.de/
