# Supplementary material for: GnRH peripherally modulates nociceptor functions, exacerbating mechanical pain
Source: Front Mol Neurosci. 2024 May 9;17:1160435. doi: 10.3389/fnmol.2024.1160435 (PMC11111891; doi:10.3389/fnmol.2024.1160435)
Supplement: Supplementary file 1 [file Data_Sheet_1.docx]

Supplementary Material

GnRH peripherally modulates nociceptor functions, exacerbating mechanical pain

**Supplementary Figures S1 to S8**

**Supplementary Figure S1. Treatment of GnRH and its analogues elicits pain-associated adverse effects.**

(A) Clinical treatment of GnRHR agonists frequently causes peripheral adverse effects associated with pain according to the reconstruction of Sider 4.1 data collection (http://sideeffects.embl.de/). (B) The reconstruction results from the Sider 4.1 data collection for GnRHR antagonists in the same manner as *(A)*.

**Supplementary Figure S2. Reconstructed information of GnRH-(+) and GnRHR-(+) DRG neuronal subclusters based on Gene Expression Omnibus data GSE59739.**

(A) Uniform manifold approximation and projection (UMAP) plots of normalized expression values of DRG neuronal subclusters in mice including neurons that contain GnRH and Calca mRNAs in single cell RNA-seq data are shown left and middle, respectively. A plot merging the left two is shown on the right. (B) UMAP plots of DRG neurons that contain GnRH and Nefh mRNAs in the same manner as in *(A)*. (C) UMAP plots of DRG neurons that contain GnRH and GnRHR mRNAs in the same manner as in *(A)*. (D) UMAP plots of DRG neurons that contain GnRHR and Calca mRNAs in the same manner as in *(A)*. (E) UMAP plots of DRG neurons that contain GnRHR and Nefh mRNAs in the same manner as in *(A)*. (F) Colors (red, GnRH or GnRHR; green, marker genes) describe the normalized expression level for each gene.

**Supplementary Figure S3. Reconstructed information of GnRH-(+) and GnRHR-(+) DRG neuronal subclusters based on Gene Expression Omnibus data GSE63576.**

(A) Uniform manifold approximation and projection (UMAP) plots of normalized expression values of DRG neuronal subclusters in mice including neurons that contain GnRH and Calca mRNAs in single cell RNA-seq data are shown left and middle, respectively. A plot merging the left two is shown on the right. (B) UMAP plots of DRG neurons that contain GnRH and Nefh mRNAs in the same manner as in *(A)*. (C) UMAP plots of DRG neurons that contain GnRH and GnRHR mRNAs in the same manner as in *(A)*. (D) UMAP plots of DRG neurons that contain GnRHR and Calca mRNAs in the same manner as in *(A)*. (E) UMAP plots of DRG neurons that contain GnRHR and Nefh mRNAs in the same manner as in *(A)*. (F) Colors (red, GnRH or GnRHR; green, marker genes) describe the normalized expression level for each gene.


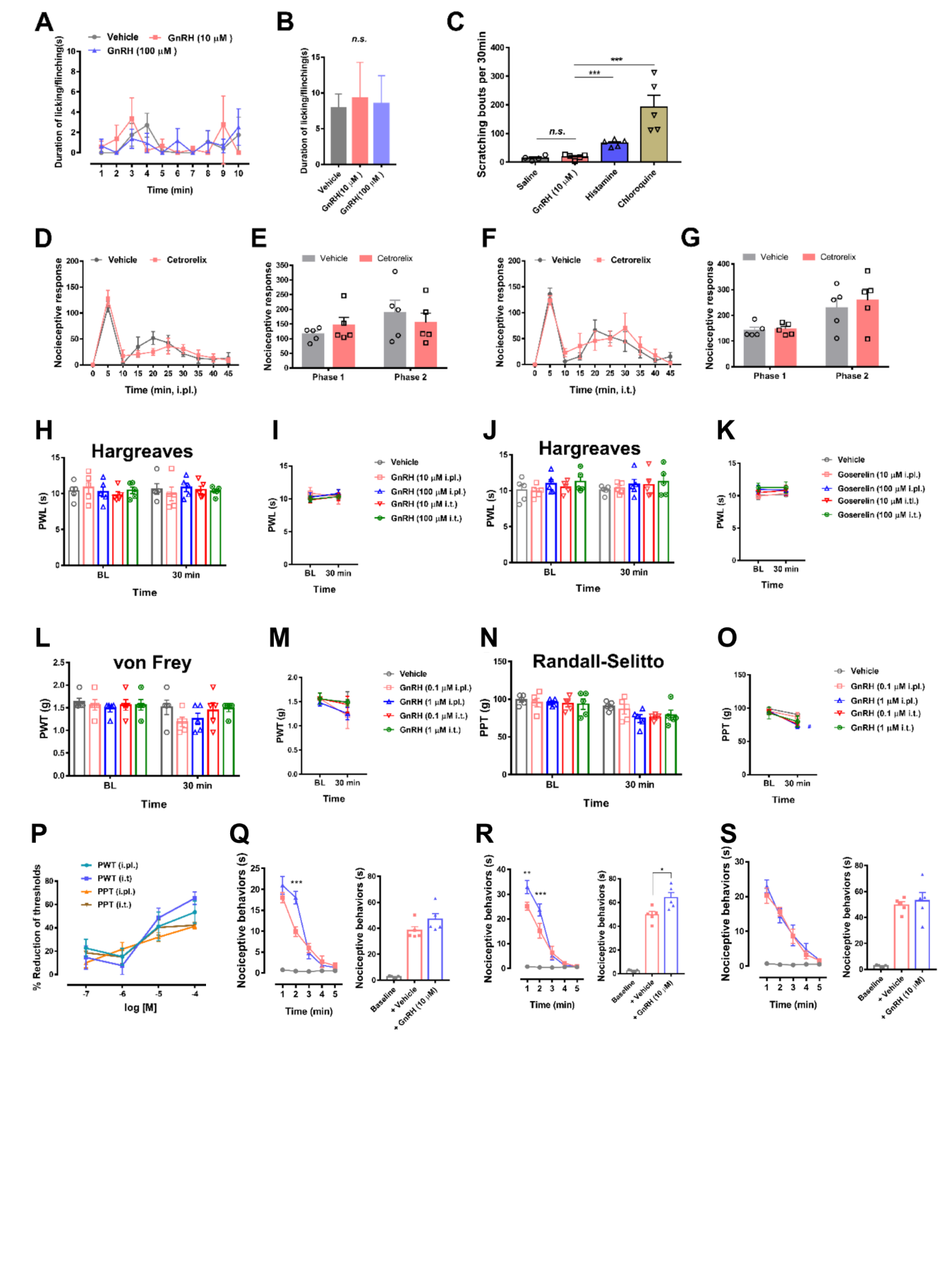


**Supplementary Figure S4. Monitoring of acute nociceptive and pruriceptive behaviors upon peripheral GnRHR activation.**

(A) Time consumption in nociceptive behaviors for 10 minutes upon intraplantarly treatment of GnRH (10 μM, red; 100 μM, blue) and vehicle (10 μl saline, gray). (B) Summarized histograms of the accumulated time engaged in the behaviors shown in *(A)*. (C) Counts in the number of scratching bouts upon treatment with GnRH (10 μM), histamine, chloroquine, and vehicle (50 μl saline). (D) Time consumption in nociceptive behaviors upon intraplantarly 5% formalin treatment. Animals were intraplantarly pretreated with vehicle (10 μl saline, gray), or cetrorelix (10 μM, red), 30 min before the formalin treatment. (E) Summarized histograms of the accumulated time engaged in early phase (phase 1) and late phase (phase 2) behaviors shown in *(D)*. (F) Time consumption in nociceptive behaviors upon intraplantarly 5% formalin treatment. Animals were intrathecally pretreated with vehicle (10 μl saline, gray), or cetrorelix (10 μM, red), 30 min before the formalin treatment. (G) Summarized histograms of the accumulated time engaged in early phase (phase 1) and late phase (phase 2) behaviors shown in *(F)*. Symbols and columns represent the mean ± S.E.M. of 5 animals (One-way ANOVA for *(B)* and *(C)* and two-way ANOVA for *(A)* and *(D)-(G)*, with Tukey’s multiple comparison test; ** p < 0.01; *** p < 0.001; n.s., not significant). (H) The paw withdrawal latencies (PWL) upon the Hargreaves heat stimulations were obtained first for baseline (B.L.) averages and were measured again 30 minutes after drug treatment. Animals were intraplantarly (i.pl.) or intrathecally (i.t.) treated with vehicle (10 μl saline), GnRH (10 μM or 100 μM). Statistic comparisons to the vehicle-treated group were performed. (I) Statistic comparisons to the averaged baseline of the same group before drug treatment were performed using the data shown in *(H)*. (J-K) Data for goserelin treatment were obtained and analyzed in the same manner as in *(H-I)*. (L) The paw withdrawal thresholds (PWT) upon the von Frey filament stimulations were obtained first for baseline (B.L.) averages and were measured again 30 minutes after drug treatment. Animals were intraplantarly (i.pl.) or intrathecally (i.t.) treated with vehicle (10 μl saline), GnRH (0.1 μM or 1 μM). Statistic comparisons to the vehicle-treated group were performed. (M) Statistic comparisons to the averaged baseline of the same group before drug treatment were performed using the data shown in *(L)*. (N) The paw pressure thresholds (PPT) upon the Randall-Selitto stimulations were obtained first for baseline (B.L.) averages and were measured again 30 minutes after drug treatment. Animals were intraplantarly (i.pl.) or intrathecally (i.t.) treated with vehicle (10 μl saline), GnRH (0.1 μM or 1 μM). Statistic comparisons to the vehicle-treated group were performed. (O) Statistic comparisons to the averaged baseline of the same group before drug treatment were performed using the data shown in *(N)*. (P) Results from threshold reduction % analyses compared to the averaged baseline of the same group before GnRH treatments at the four doses presented in Figures 3 and L-O. (Q) Time course of the duration of 0.1% capsaicin-induced nociceptive behaviors in mice. Animals were intraplantarly pretreated with vehicle (10 μl saline, red), or GnRH (10 μM, blue), 30 min before the capsaicin injection. Total durations of nociceptive responses for 5 min are quantified in the right histogram. (R) Time course of the duration of 10 mM cinnamaldehyde (CA)-induced nociceptive behaviors in mice. The effects of GnRH were monitored in the same way as in *(Q)*. (S) Time course of the duration of 140 mM KCl-induced nociceptive behaviors in mice. The effects of GnRH were monitored in the same way as in *(Q)*. Five animals were used for each data point in *(Q) – (R)* (One-way ANOVA with Tukey’s multiple com-parison test. * p < 0.05, ** p < 0.01, *** p < 0.001).


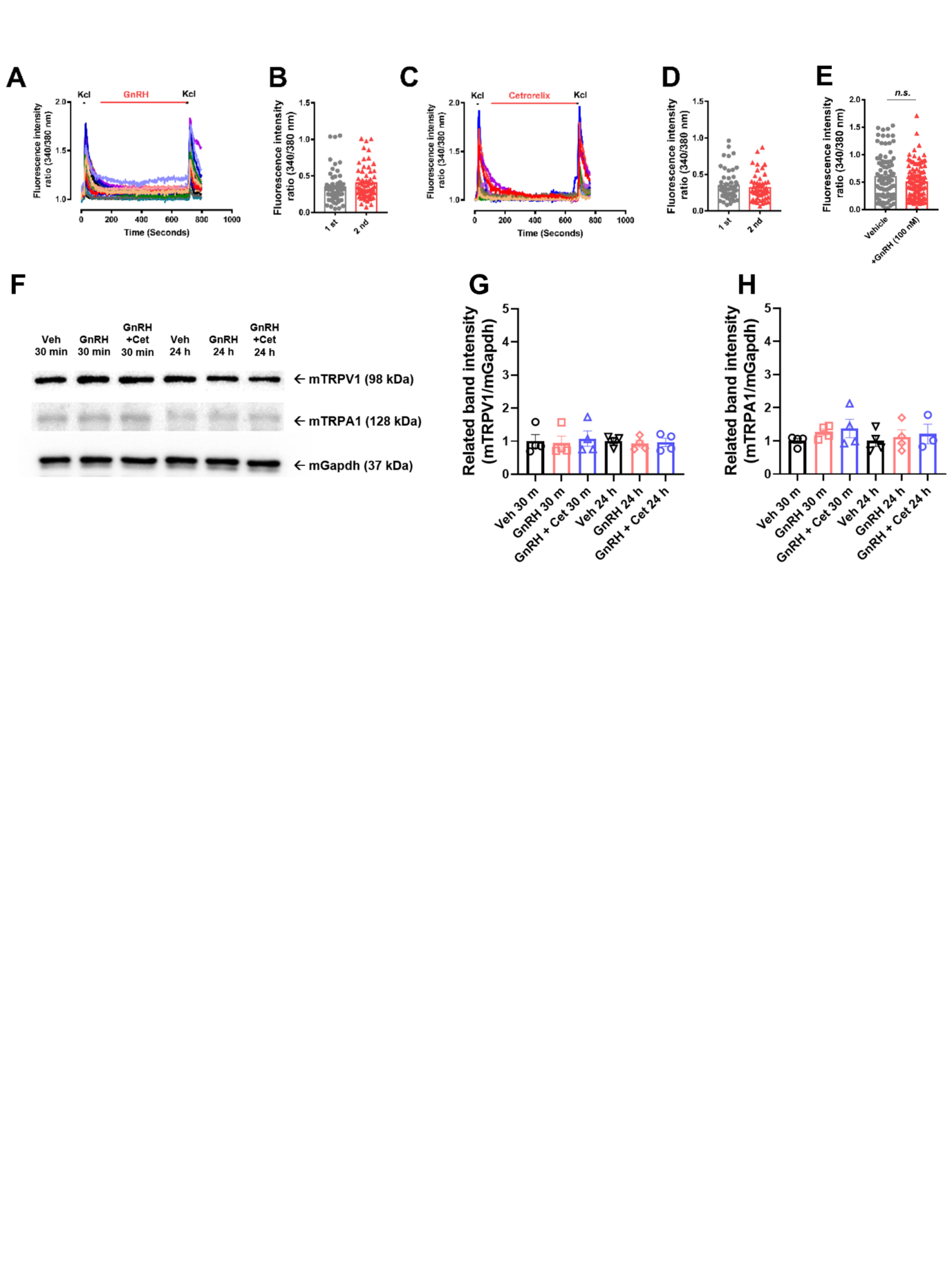


**Supplementary Figure S5. GnRH and cetrorelix failed to alter intracellular Ca^2+^ levels by KCl-induced depolarization in DRG neurons and TRPV1 and TRPA1 expressions were tolerant to GnRH treatment in DRG neurons.**

(A) Representative traces for intracellular Ca^2+^ monitoring when 100 nM GnRH or 60 mM KCl was applied in Fura-2 Ca^2+^ imaging experiments using cultured murine DRG neurons. (B) Collection of the amplitudes of highest Ca2+ peaks upon 1st and 2nd KCl applications in *(A)*. (C) Representative traces for intracellular Ca^2+^ monitoring when 10 μM cetrorelix or 60 mM KCl was applied in Fura-2 Ca^2+^ imaging experiments using cultured murine DRG neurons. (D) Collection of the amplitudes of highest Ca2+ peaks upon 1st and 2nd KCl applications in *(C)*. (E) Collection of the amplitudes of highest Ca^2+^ peaks upon KCl applications with (right) or without 24 h pre-incubation of 100 nM GnRH (left) resented in Figure 4. (F) Representative western blot results of TRPV1 and TRPA1 proteins from DRG neurons under GnRH exposure with or without cetrorelix (Cet). (G-H) to those from vehicle treatment (Veh). The data in *(G) – (H)* were expressed as the mean ± S.E.M. and statistical significance was not detected in One-way ANOVA followed by Bonferroni’s post hoc test.

**
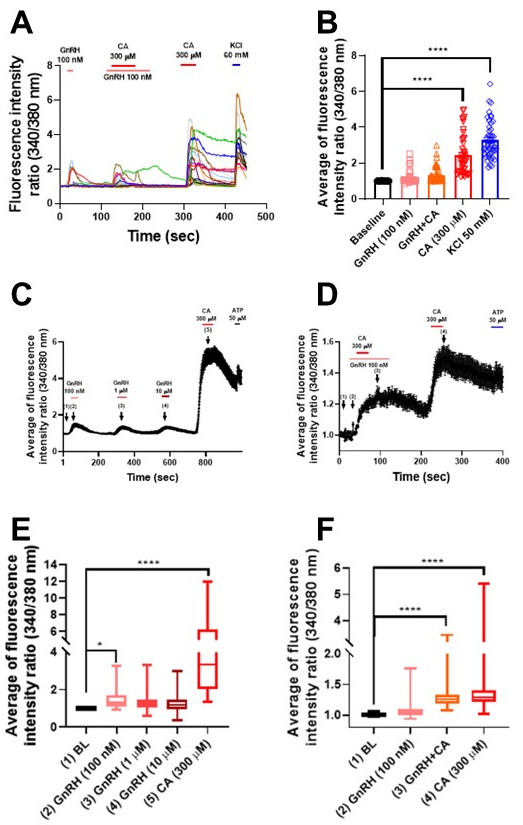
**

**Supplementary Figure S6. GnRH shows a weak partial antagonistic activity for TRPA1 activation.**

(A) Fluorescence was mildly elevated upon 100 nM GnRH treatment in a subset of cultured DRG neurons in Fura-2 Ca^2+^ imaging experiments. Responses upon cinnamaldehyde (CA) treatment, presumably mediated by TRPA1 activation, were blunted by the presence of GnRH. (B) Average fluorescence intensities of their peak responses in GnRH- or CA-responder neurons among KCl-responder neurons. GnRH responses were not statistically significant. (C-D) Average fura-2 fluorescence upon treatments of three different doses of GnRH and CA *(C)*, and upon co-treatments of GnRH and CA *(D)* in murine TRPA1-transfected HEK293 cells. (E-F) Fluorescence intensities of GnRH- and CA-responder transfected cells were averaged and the intensities caused by GnRH were largely ignorable. All experiments were triplicated and each column represents the mean ± S.E.M. (* p < 0.05, **** p < 0.0001, One-way ANOVA followed by Tukey’s post hoc test).


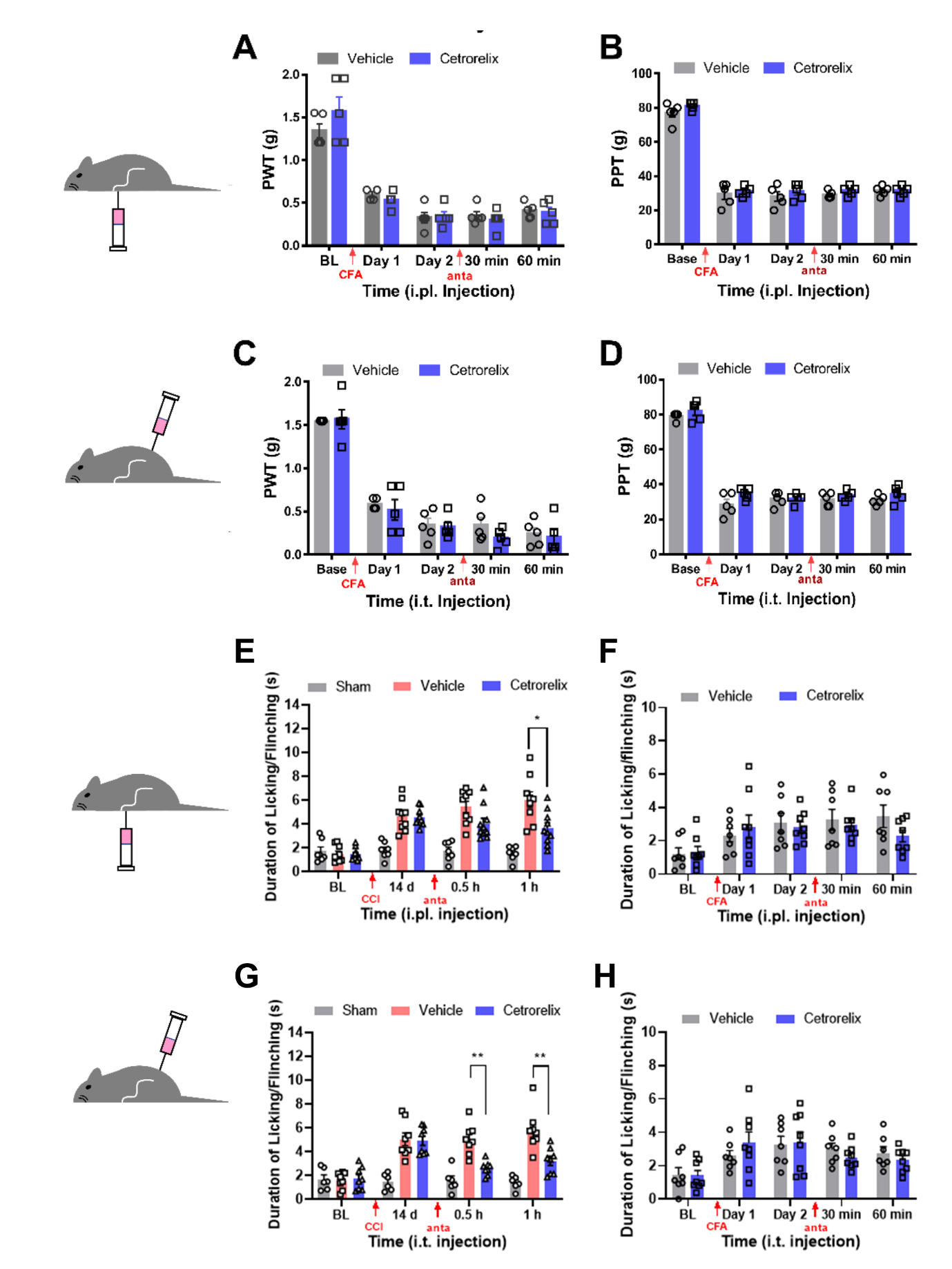


**Supplementary Figure S7. GnRH did not contribute to inflammatory pain but to cold hypersensitivity in neuropathic pain models.**

(A) Time course of von Frey thresholds in complete Freund’s adjuvant (CFA)-inflamed mice. Animals were intraplantarly treated with vehicle (gray) or cetrorelix (10 μM, blue) in the ipsilateral hind paws 48 h after CFA injection. (B) Time course of Randall-Selitto thresholds in CFA-inflamed mice. Animals were intraplantarly treated with vehicle (gray) or cetrorelix (10 μM, blue) in the ipsilateral hind paws 48 h after CFA injection. (C) Time course of von Frey thresholds in CFA-inflamed mice. Animals were intrathecally treated with vehicle (gray) or cetrorelix (10 μM, blue) 48 h after CFA injection. (D) Time course of Randall-Selitto thresholds in CFA-inflamed mice. Animals were intrathecally treated with vehicle (gray) or cetrorelix (10 μM, blue) 48 h after CFA injection. (E) Time course of pain behavioral duration upon acetone stimulation in chronic constriction injury (CCI). Animals were intraplantarly treated with vehicle (pink) or cetrorelix (10 μM, blue) in the ipsilateral hind paws 14 days after CCI surgery. (F) Time course of pain behavioral duration upon acetone stimulation in CFA-inflamed mice. Animals were intraplantarly treated with vehicle (gray) or cetrorelix (10 μM, blue) in the ipsilateral hind paws 48 h after CFA injection. (G) Time course of pain behavioral duration upon acetone stimulation in CCI. Animals were intrathecally treated with vehicle (pink) or cetrorelix (10 μM, blue) in the ipsilateral hind paws 14 days after CCI surgery. (H) Time course of pain behavioral duration upon acetone stimulation in CFA-inflamed mice. Animals were intrathecally treated with vehicle (gray) or cetrorelix (10 μM, blue) in the ipsilateral hind paws 48 h after CFA injection. Each column represents the mean ± S.E.M. (* p < 0.05, ** p < 0.01, Two-way ANOVA followed by Tukey’s post hoc test).


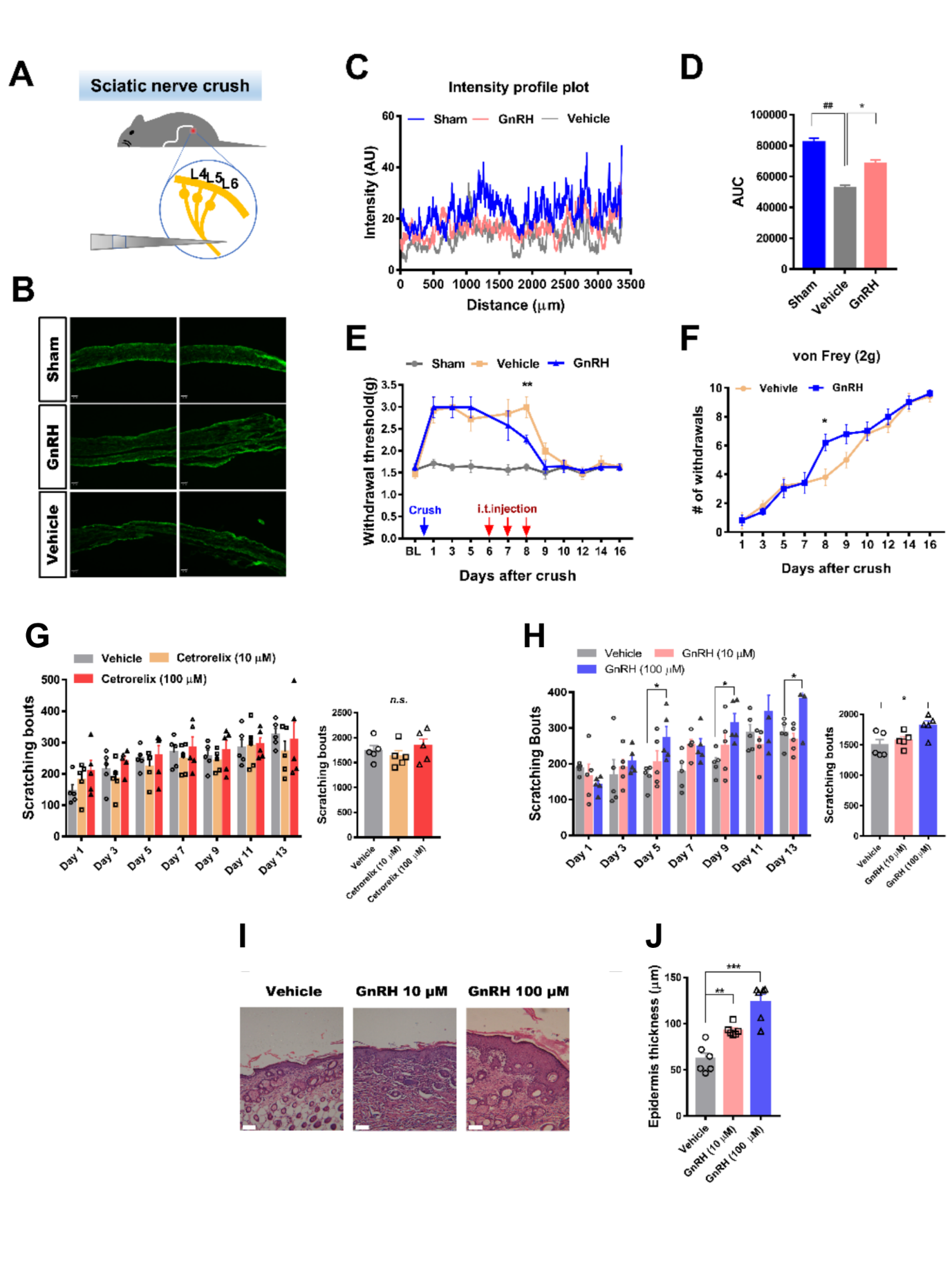


**Supplementary Figure S8. Contribution of GnRHR activation to peripheral nerve restoration and to pathologic itch-induced scratches.**

(A) Schematic drawing of sciatic nerve crush. The sciatic nerve was exposed and carefully freed of connective tissue and fully crushed for 15 s using forceps. (B) Representative NF200 immunostaining of sciatic nerves 8 days after crush injury in intrathecally vehicle-treated and 10 μM GnRH-treated mice and without (sham) a conditioning injury (Scare bars, 100 μm). (C) Profiles of intensities of NF200 immunofluorescence are shown in (B). Intensities were quantitated using ImageJ. (D) The area under the curve (AUC) of the intensity profile shown in *(C)*. (E) Time course of von Frey thresholds in mice that received sciatic nerve crush. Mice were intrathecally treated with 10 μM GnRH on days indicated by red arrows. (F) Time course of the sensitivities upon 2 g mechanical stimulations in vehicle-treated and GnRH-treated mice after sciatic nerve crush. The numbers of brisk withdrawals out of 10 stimulations of 2 g von Frey filaments were quantitated. Two-way ANOVA with Tukey’s multiple com-parison test was performed. (G) Spontaneous scratching behaviors of DNCB-treated mice for 1 h, once every two days when vehicle or cetrorelix was intradermally treated on the pathologic regions (left). Cumulative time engaged in scratching behaviors is summarized in the histogram (right). (H) Spontaneous scratching behaviors of DNCB-treated mice for 1 h, once every two days when vehicle or GnRH was intradermally treated on the pathologic regions (left). Cumulative time engaged in scratching behaviors is summarized in the histogram (right). (I) Representative photographs of hematoxylin and eosin (H&E) staining of DNCB-spread dorsal skin with or without GnRH injection on day 13. (J) Quantitated epidermal thickness of DNCB-spread dorsal skin with or without GnRH injection.
